# The microgenesis of the watercolor effect

**DOI:** 10.3389/fpsyg.2013.00702

**Published:** 2013-10-17

**Authors:** Adam Reeves, Baingio Pinna, Felix Roxas

**Affiliations:** ^1^Department of Psychology, Northeastern UniversityBoston, MA, USA; ^2^Department of Architecture, Design and Planning, University of Sassari at AlgheroSardinia, Italy

**Keywords:** color, illusions, microgenesis, masking noise, matching

## Abstract

The “watercolor effect” is the wash of illusory color that fills in between two enclosing bichromatic contours. We studied the microgenesis of this illusion by varying the duration of the eliciting stimulus (a yellow/purple contour outlining the Mediterranean Sea) and by varying the duration of a blank interval from stimulus offset to an after-coming mask (the ISI). The illusory wash was rated in strength and also matched to a comparison disk of adjustable color but similar luminance. Results indicate that the watercolor effect grows rapidly as stimulus duration is increased to 100 ms and then grows much more slowly. Increasing the ISI beyond 10 ms had no effect, suggesting that the wash arises only during stimulation. Participants who recognized that the bounding contour depicted the Mediterranean reported twice as strong an illusory effect as those who did not, indicating that visual long-term memory modulates the watercolor effect despite the rapidity of its generation.

## Introduction

Outlining a shape with a single color on a uniform gray field or gray paper normally affords perception of the outline by itself, with no filling-in of the enclosed area. However, if the shape is drawn as a bichromatic contour, with, for example, yellow on the inside and purple on the outside, an illusory wash of pale yellow appears to fill in the enclosure—the “watercolor effect” of Pinna (Pinna, 1987, 2008; Pinna et al., 2003; Pinna and Reeves, 2006). Conversely, if the yellow is on the outside, the illusory wash spreads outwards from the contour to the edge of the display, the central region remaining gray. Both effects are quite salient with an outline of the Mediterranean Sea (Pinna, 1987, 2008; Spillmann et al., 2004; Pinna and Mariotti, 2005), and we employed this stimulus again in the current research. Illusory watercolor filling-in generally occurs only on the weaker side of the bichromatic contour, that is, the side with lower luminous contrast relative to the background (Devinck et al., 2005; Pinna, 2005). In our display, yellow has lower apparent contrast than purple when presented on a gray field.

The watercolor effect is compelling; observers are certain that the filled-in color they see is genuine, not an illusion (Pinna, 1987, 2008). The illusion has several important properties related to filling-in process (Walls, 1954; Pinna et al., 2001), namely: it is independent of spatial extent, being the same for big as for small shapes; it is spatially uniform, being seen with equal strength across the entire enclosed region, not just at the edges; it is essentially independent of retinal eccentricity, not requiring that the contour be fixated; and it does not decrease over time. These properties agree with some of those deduced from filling-in between retinally-stabilized edges (Krauskopf, 1963) and for brightness perception (Paradiso and Nakayama, 1991), but not with those of some other classic “filling-in” phenomena, such as filling in across a scotoma, whose spatial extent is restricted. Although the wash appears desaturated, it creates a strong grouping, such that the filled-in area becomes figural. Indeed, the grouping due to the illusory wash is stronger than the Gestalt factors (Koffka, 1935) proximity, similarity, and good continuation, when tested in a cue conflict paradigm (Pinna, 2010), and is stronger than the grouping produced by a matched real color (von der Heydt and Pierson, 2006). Curiously, the two colors in the bichromatic contour can be slightly separated and yet still induce a watercolor effect (Pinna et al., 2001; Pinna and Deiana, submitted). Neither of these two latter properties are representative of filling-in *per se*.

These properties of the watercolor effect are compatible with general principles underlying the distinction between the featural “FCS” system and the boundary “BCS” system advanced by Grossberg and Mingolla (1985). In that theory, spatially-localized BCS signals constrain the flow of visual features (colors, textures, and light/dark signals) which “fill-in” between the boundaries. The BCS/FCS signals are data-driven, arising in a bottom-up manner, but in our understanding, the featural landscape thus generated has the potential to resonate with top-down long-term visual memory to support recognition and to yield consciously perceivable visual objects. Illusory, spatially-uniform, eccentricity-independent illusory color washes between bounding contours can be explained in this fashion (Pinna and Grossberg, 2005). That a real (non-illusory) wash of equivalent saturation does not form as strong groupings as the watercolor stimulus (Pinna, 2008, 2010) suggests that such a resonance may be needed to sustain the watercolor effect, whereas in the case of a real wash, such a resonance is not as necessary since the supporting feature (color) is bottom-up driven.

In the present study we asked two questions about how the illusory effect is generated. Since the wash seems to appear along with the contours, we expected the time course of its generation—its so-called microgenesis (Werner, 1956)—to be relatively fast. In the BCS/FCS theory, filling-in is initially determined by automatic, fast, low-level processes (Grossberg, 1997). Pinna et al. (2001) reported that the illusion was visible at the shortest presentation time permitted by their equipment, namely, 100 ms. Huang and Paradiso (2008) reported that filling-in within monkey V1 cells takes on average 80 ms and is complete by 200 ms, taking longer for longer distances. We note that the microgenesis of the wash, though fast, is not likely to be as fast as that of the bounding contours themselves, as predicted since BCS processing is prior to FCS filling-in. Second, we asked if there exists a top-down, cognitive contribution to the illusion. A rapid, low-level, feed-forward visual filling-in process would not be influenced by knowledge of the shape of the bounding contour, but in the BCS/FCS system, top-down, memory-driven resonances could strengthen the boundary and increase the illusion. The version of the illusion we used was a sketch of the Mediterranean Sea, and we simply asked whether participants who recognized the Sea would have a stronger illusion that those who did not. (As the eliciting stimulus is constant, this method was preferred to the alternative of comparing well-known and nonsense objects, which confounds knowledge with physical shape differences.) Pinna (2012) has argued that from a developmental perspective that visual object formation occurs according to the following sequence: contours, color, shading, and lighting. To the extent that this also applies to microgenesis, we expect to see the same sequence develop as stimulus duration is increased. The present research, by using the outline of the Mediterranean Sea, addresses the first of these only; other stimuli will be needed to study the development of shading and lighting.

The first Experiment adopted a matching procedure to measure the extent of the watercolor effect in continuously-presented stimuli objectively, and also to determine the chromaticity of a standard or “anchor” stimulus used for a subjective, rating procedure in the second experiment. This rating procedure allowed us to measure the time-course of development of the illusion with reasonable efficiency in both the second and third experiments (the latter involved a variant rating procedure); only the duration of the stimulus was varied. The fourth employed backward masking to control stimulus persistence, analogous to the use of backward masking to control brightness filling-in by Paradiso and Nakayama (1991).

## Materials and methods

### Participants

These were undergraduates of Northeastern University with normal vision, 20/20 or better visual acuity, and normal color vision as determined by Ishihara's (1965) pseudo-isochromatic plates, aged between 17 and 24 years old. The number of participants in experiments 1, 2, 3, and 4 was 17, 34, 12, and 30, respectively. All participants signed an informed consent that had been vetted by Northeastern's IRB. They received course credit, but were free to leave the experiment at any time, even before completion.

### Stimuli and apparatus

**Figure 1:**
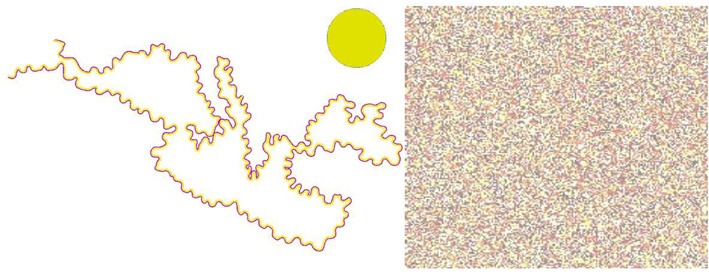
**Left:** Yellow In (Yin) stimulus configuration. **Right:** texture mask used in *masking*.

Two printed bichromatic outlines of the Mediterranean Sea and its surrounding countries, used in Pinna's (1987, 2008; see also Spillmann et al., 2004; Pinna and Mariotti, 2005) original study, were scanned as pic files and presented as 33 × 17 cm images on a calibrated Sony 19″ diagonal cathode-ray display monitor (see Figure 1, left panel). Viewed from 63 cms, the outlines subtended 27.7 × 15.1 degree of visual angle and covered roughly the lower two-thirds of the monitor screen (whose width was 33 degree, height 26 degree). A filled disk of 4.7 degree dia. served as a comparison (see Figure 1, left panel, upper right corner, where the disk is portrayed with a strong yellow). The center of the disk was 7.4 degree from the top of the screen and 8.5 degree from the right-hand edge. The disk was separated from the nearest point on the outline of the Sea by 2.2 degree. The watercolor and comparison stimuli appeared on a uniform gray field of 116 cd/m^2^ which covered the entire 19″ display. The bi-chromatic outline of the Sea was drawn with two flanked lines of yellow (which subtended 6 m min arc) and purple (5 min arc). One figure had purple on the outside and yellow on the inside of the contour (Yellow In, or Yin), and the other figure had the reverse (Yellow Out, or Yout); Figure 1 portrays the Yin stimulus.

The monitor was driven by a Cambridge Research Systems VSG-5 card programmed in Matlab V.6 and run under Windows XP. The VSG card provides accurate timing of display frames when run repeatedly in “movie” mode, as confirmed with a counter triggered by a photodiode: every 10 ms frame was timed correctly over a 20 min. calibration period. The nominal chromaticity of the gray field was (0.290, 0.300) in CIE (*x, y*) co-ordinates as recorded with a calibrated Cambridge Research Systems colorimeter. This gray point is very slightly bluer than most standard daylights—for example, Illuminant C is (0.310, 0.316). This gray was chosen as being red/green neutral and having no yellow in it, to ensure that any perceived yellow was illusory. The (x, y) chromaticities of the yellow and purple lines used to draw the stimuli were (0.480, 0.500) for yellow and (0.301, 0.120) for purple. Their luminances were 141 cd/m^2^ and 53 cd/m^2^ respectively. To make these measurements, the chromaticities were duplicated over areas large enough to fill the aperture of the colorimeter.

## Experiment 1: matching

Matching experiments were run to obtain an objective or “Type A” (Brindley, 1960) measure of the strength of the illusory wash with the Sea. Research using phenomenological ratings or other verbal descriptions (“Type B” measures) has established conditions in which the illusion is seen, but not its precise extent. Type “A” matches of real to illusory colors in a variant watercolor stimulus due to Pinna et al. (2003), namely, irregular outline squares with purple and orange bichromatic contours, produced watercolor effects of 3% of the distance to the inducing orange in a CIE uniform color space (von der Heydt and Pierson, 2006) and of 5.6% in a near replication (Devinck et al., 2005). Our participants similarly matched the color of the illusory area to that of a real comparison stimulus. We permitted almost continuous variation of the color coordinates of the matching stimulus, as did Devinck et al. (2005), rather than employing a discrete series of stimuli, as in von der Heydt and Pierson (2006), as the effect was expected to be small.

In an ideal type A match, the two areas, once matched, are indiscriminable. Type A matches have been critical to visual science since they imply identity of the signals from the two areas at all sites in the visual pathway subsequent to the first site at which identity occurs. Such matches have been invaluable in analyses of higher levels of the visual pathway (as for example in color induction) as well as in classical studies of receptor properties. Some researchers have qualified this picture, since in the case of asymmetrical matches (e.g., between lights viewed under different illuminants), subjects feel they should be able to improve on even their best matches (e.g., Brainard et al., 1997), indicating that the signals from the two areas are not always indiscriminable. In our experiments the scene illumination was constant and the illusory wash appeared as a surface color (the illusion being cognitively impenetrable), not as a trick of the lighting, so Type A matches were possible. If signals from matched real and illusory areas are indeed identical, the illusion should be cancelled by a real complementary stimulus of the same magnitude, as found by Devinck et al. (2005).

To make matches, the 17 participants had mouse control over the two chromatic co-ordinates (*x*, *y*) of a uniformly filled comparison disk, positioned outside the illusory area, of fixed size (3 degree of visual angle) and fixed luminance, but variable hue and saturation. The disk was given an arbitrary chromaticity (0.2 < *x* < 0.4; 0.2 < *y* < 0.4) at the start of each trial^1^, and the participant was asked to adjust its color to match that of the wash. Small mouse movements initially made large differences in disk color, but the left mouse button could be pressed at any time to halve the step size, so the participant could narrow in on the best match. Best matches were signaled to the computer by a right button press, ending each trial. Note that the illusory stimulus remained present, and physically unchanged, throughout the matching procedure. Fortunately the illusion is stable and does not fade while the matches are being made in this painstaking manner.

There were four conditions. In the two *complementary* conditions, participants matched the comparison disk to the gray inside region when the stimulus was Yellow Out (Yout), or to the gray outside region when the stimulus was Yellow In (Yin). We anticipated that these matches would on average equal the chromaticity of the gray field, given that the bichromatic contour exerts an illusory effect only in the opposite direction. In the two *illusion-generating* conditions, participants matched the comparison disk to the gray inside region when the stimulus was Yin, or to the gray outside region when the stimulus was Yout.

Participants were run for 3 to 5 blocks of 10 trials in each condition in each block, for a total of 40 trials per block. Thus 30, 40, or 50 matches were made per participant per condition. The order of conditions in each block was random, except that 9 of the participants began with illusion matches and 8 with complementary matches. The participants who ran fewer than 5 blocks took extra time per trial, there being no explicit time pressure.

Although the matching instructions were understood and making matches to gray was found to be easy, making matches to the illusion was not easy. Participants reported that the color of the wash, though perceptually salient, was hard to capture, and the ultimate matches were often felt to be “as good as possible” rather than exact. In this respect the matches were not pure Type A, but like the asymmetrical matches of Brainard et al. (1997).

### Results (matching)

**Figure 2:**
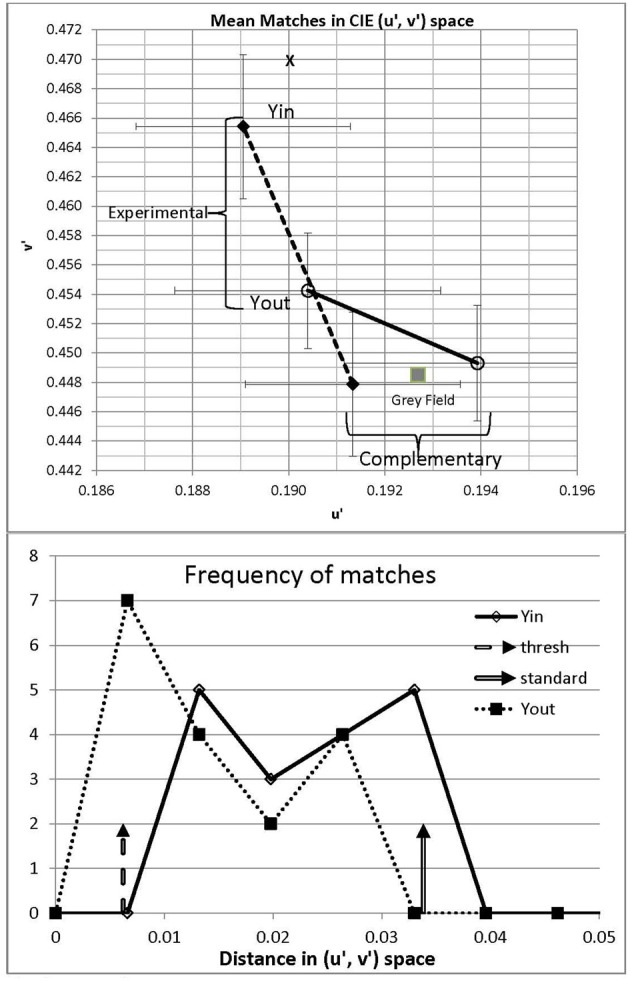
**Upper panel:** Mean matches in Experiment 1 for Yin and Yout configurations. Matches were made to the illusory area and also to the complementary gray area as a control; the actual gray field is noted by a filled square for comparison. Error bars show ±1 standard deviation of trial-to-trial matches in the *u*′ and *v*′ directions, averaged over 17 participants. The spectrum locus lies outside this plot: the dominant wavelength corresponding to Yin, 565 nm, would be located at *u*′ = 0.177, *v*′ = 0.573. **Lower panel:** frequency polygons of mean matches from the 17 participants in Yin and Yout, plotted against distance in (*u*′, *v*′) space to the matched gray. Threshold for a visible change from gray to yellow is marked by the left-hand broken vertical arrow. The standard used in Experiment 2 is marked by the right-hand vertical arrow.

Matches (Table 1) were transformed to (*u*′, *v*′) space as this co-ordinate system more nearly approaches a uniform chromaticity scale than does (*x, y*) (Wyszecki and Stiles, 1982). Matches for the two *complementary* areas, where no illusion was anticipated, averaged (*u*′ = 0.191, *v*′ = 0.448) for the gray outside region of Yin and (0.194, 0.449) for the gray inside region of Yout (see the lower right corner of Figure 2). These values deviated slightly from the actual gray field (0.193, 0.449), but not significantly as the error bars overlapped the gray field coordinates.

**Table 1:** **Matches in complementary and Experimental regions**.

	**Yin**		**Yout**	
	***u*^′^**	***v*^′^**	***u*^′^**	***v*^′^**
**COMPLE**				
Mean	0.1913	0.4479	0.1939	0.4493
SE-bet	0.0065	0.0072	0.0096	0.0080
SE-w	0.0022	0.0049	0.0032	0.0049
**EXPTAL**				
Mean	0.1891	0.4654	0.1904	0.4543
SE-bet	0.0100	0.0063	0.0082	0.0121
SE-w	0.0028	0.0037	0.0028	0.0039

Consistent with their being a color shift in the *illusion* areas, the chromaticity matched to the central gray region averaged to (*u*′ = 0.189, *v*′ = 0.465) in Yin and (0.190, 0.454) in Yout. The illusion in Yin represents 13% of the distance in (*u*′, *v*′) space from the white point to a monochromatic yellow of wavelength 565 nm; the corresponding distance in Yout being 6%. These distances indicate fairly desaturated illusory colors, but as the threshold for discriminating white from yellow is 3.1% in (*u*′, *v*′) space (Wyszecki and Stiles, 1982), the illusory yellow in Yin would be visible to the average participant, being four times threshold.

The illusion generated by the Yin version of the Sea, being 13%, was clearly stronger than that 5.6% generated by the variant watercolor illusion used by Devinck et al. (2005) and the 3% reported by von der Heydt and Pierson (2006). Such a large difference awaits explanation; part of the reason may be the recognizability of the Sea, to which we will turn in Experiment 2.

Individual differences could be ascertained fairly reliably, based on the 30, 40, or 50 trials run in each condition. Participants were highly reliable in their settings, their standard deviations averaging just *u*′ = 0.0028, *v*′ = 0.0037 over participants in Yin (the error bars shown in Figure 2). Euclidean distances in (*u*′, *v*′) space from each participant's white point to his or her average illusory chromaticity in Yin varied widely, from 0.0077 to 0.032, across the 17 participants. It is likely that 15 of them experienced illusions, these distances being more than 3 standard deviations above threshold, but the two participants with the smallest distances, 0.0077 and 0.0087, may not have done so. Across participants, the magnitude of the illusion in Yin did not correlate significantly with that in Yout [*r* = 0.285, *t*_(15)_ = 1.16, n.s.], suggesting independent processing of the different regions.

The results of the matching experiment show that the Watercolor effect can be measured using a matching procedure, and indicate that the effect is reliably greater than threshold for nearly all participants. The effect is about twice as large in Yin than in Yout, consistent with the filling-in of an enclosed area being stronger than spreading outwards into an indefinite region. In addition, the neutrality of the complementary (non-illusory) areas was confirmed. The matches were of high quality, the standard deviations being low. However, some participants reported that the illusory area could still be discriminated from the comparison at the match point, so these were not all true Type A matches, either because the luminance of the matching stimulus could not be adjusted, or because the color of the illusory wash differs from that of the matching stimulus in some other, qualitative, manner.

## Experiment 2: rating *Re* a physical anchor

To obtain systematic data as a function of stimulus duration, which required many more data points than we obtained in matching, we used a faster rating procedure.

### Procedure

The same watercolor stimuli were used as in Experiment 1. The disk used for matching in Experiment 1 now provided a physical anchor or standard for the rating scale. The disk was a fixed pale yellow shown by the cross in Figure 2 (at *u*′ = 0.190, *v*′ = 0.470). Participants were first shown a black and white paper version of the stimulus to test their recognition of the Mediterranean Sea from its outline. Some could not do so, not being of European origin, unlike Pinna's Italian observers. After the experiments were over, those who had not recognized the Sea were asked if they had done so during the experiment, to check for delayed recognition. This was the case for just four observers, whose data were folded in with those who initially recognized the Sea; this did not change the pattern of results. Participants now only rated the illusory regions, not the complementary areas, since participants in Experiment 1 had matched the complementary areas to gray. (As a check, though, every participant also rated the complementary areas once at the start of de-briefing, and they did rate them as 0, meaning gray.)

All participants were instructed to rate the strength of the color wash from 0, or none, to 4, the rating assigned to the standard, with 1 designated as a just-visible illusion, and 2 and 3 to be spaced as equally as possible between 1 and 4. The same standard was used in all conditions for all participants. Ratings 2 and 3 were not anchored but left up to the participants' understanding of “equally-spaced.” (Here, strength implies the saturation and perhaps brightness of the yellow wash, as the illusion did not affect hue.) The standard could be requested at any time between trials, upon the participant's key press. After initial practice, such requests became infrequent as participants mostly relied on memory. In debriefing, participants reported understanding the rating task and being able to use the scale as requested. Although the standard was more saturated than the mean match made in Yin by every participant in Experiment 1 (see Figure 2, lower panel), nevertheless, due to variability over trials in illusion strength, participants with strong illusions might have wanted to use a rating of “5” on occasion. We had abandoned this rating level in pilot work as it was rarely used, but the highest mean ratings, those obtained at the longest durations, may yet have been somewhat truncated.

Participants viewed eight different durations of the stimulus, namely, 10, 20, 30, 100, 300, 1000, 3000, and 10000 ms. Each duration was presented four times in random order, for a total of 32 trials per participant per stimulus. After each stimulus presentation, participants gave their rating. Half the participants rated the Yellow In (Yin) stimulus first, and then the Yellow Out (Yout); the order was reversed for the other half. They rated the *illusory* areas, that is, they rated the central, bounded, region in Yin and the region outside the bounding contour in Yout. Each participant rated four trials of Yin and four trials of Yout, at each duration.

### Results and discussion (ratings; fixed anchor)

Confirming Pinna (1987, 2008), not one of our 34 participants realized that the wash was illusory. (Many asked us to prove this was so: we complied, after debriefing, by blocking off sections of the bounding contour.) The illusion is cognitively impenetrable even when participants are rating or matching the illusory stimulus multiple times for an entire hour.

Participants were separated into those who recognized and those who did not recognize the Mediterranean Sea from its outline, as determined during de-briefing. As 16 recognized and 18 did not, the sizes of the two groups were comparable. (*En passant*, we note that the data of the participants who reported feeling bored by the experiment did not differ systematically from the rest, so results were collapsed over interest level.)

The four ratings of each condition and duration provided by each participant were averaged and taken as the input for all statistical calculations and graphs. There were large individual differences, whose causes are unknown: some participants needed just 3 frames (30 ms) to reach their maximum rating, while others needed 100 frames (1 s) in the “recognize” group, or as much as 500 frames (5 s) in the “unrecognized” group.

**Figure 3:**
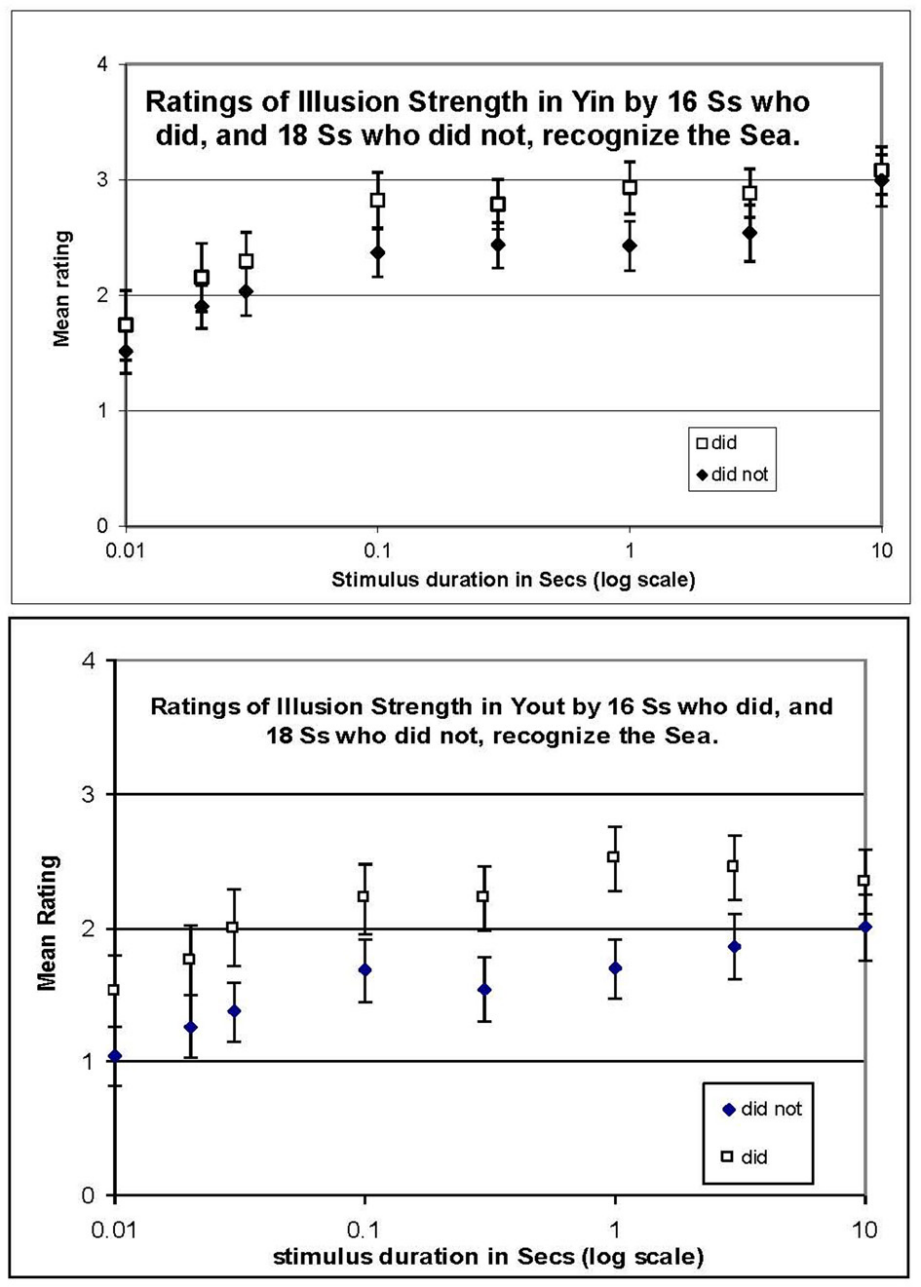
**Mean ratings in Experiment 2 with a single fixed anchor stimulus (the cross in Figure 2) rated “4,” and no illusion (gray) rated “0,” for Yin (top panel) and Yout (bottom panel), plotted against stimulus duration in log seconds**. Participants who did or did not recognize the Sea are plotted separately. Error bars show ±1 standard error of each mean.

Mean ratings over participants are plotted in Figure 3 with error bars based on standard errors across participants. It should be kept in mind that the error bars are small due to the large number of participants, not to lack of systematic individual differences. Despite individual differences, the results were consistent in that the illusion strength in Yin was always rated as greater than in Yout, and in every participant, the illusion strengthened with duration, so the mean data are in this sense representative.

Plots of mean rating vs. stimulus duration in Figure 3 show a progressive increase in illusion magnitude as stimulus duration was increased. Logarithmic time axes were used in graphs to avoid compressing the data at short durations; however, all reported growth rates are from regressions of mean ratings against linear time. The data appear to fall into two regimes, with a fast rise at durations below 0.3 s followed by a slow rise thereafter. That is, mean ratings in Yin (Figure 3, top panel) rose rapidly from 0.01 to 0.3 s, at 1.0 rating unit per tenth of a second (1.0/100 ms) for recognizers, and 0.77/100 ms for non-recognizers. In contrast, from 0.3 to 10 s, ratings rose by only 0.03/s for recognizers and by 0.06/s for non-recognizers (n.s.). Data for Yout (bottom panel) were similar, in that ratings also increased rapidly from 0.01 to 0.3 s, at 0.64/100 ms for recognizers, and 0.88/100 ms for non-recognizers (*r*^2^ = 0.77 and.88, respectively), while from 0.3 to 10 s ratings increased at a rate of only 0.02/s for both participant groups (n.s.). It is also clear from the plots in Figure 3 that ratings were higher for those who did recognize the Sea than those who did not, at each stimulus duration.

**Figure 4:**
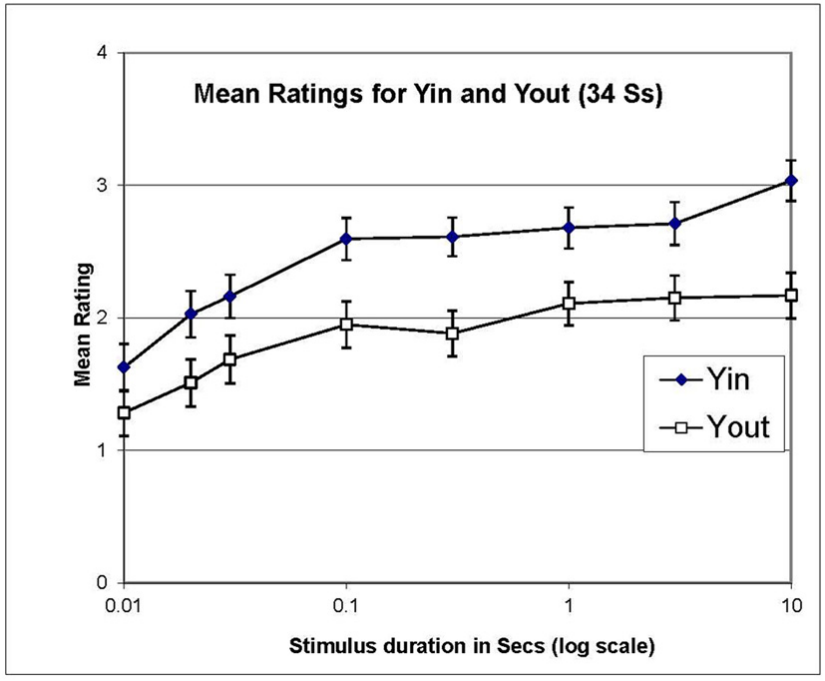
**Mean ratings of illusion strength in Experiment 2, for Yin and Yout, averaged over recognizers and non-recognizers and plotted against stimulus duration in log s**. Error bars show ±1 standard error of each mean.

Averaging ratings over both recognizers and non-recognizers, ratings for Yin were higher than those for Yout, consistent with the matching data from Experiment 1, as is made clear in Figure 4. The difference between the ratings in Yin and Yout increased slightly at longer stimulus durations, but it is notable that a difference is evident even with a stimulus duration of only one frame (0.01 s).

## Experiment 3: relative rating

In Experiment 3, 12 new participants were trained to use a relative rating scale, in which “4” represented the illusion when the Sea was continuously presented, both in Yin and, separately, in Yout. There was no fixed comparison disk. Other ratings were designated as before. In this manner we could compare the growth of Yin and Yout ratings over time, while normalizing out the differences between their absolute magnitudes that appeared in Experiment 2. Only those who recognized the Sea were run. The procedure was the same as in Experiment 2, except that the longest duration was 5 s.

### Results and discussion (ratings; relative to 4.0)

**Figure 5:**
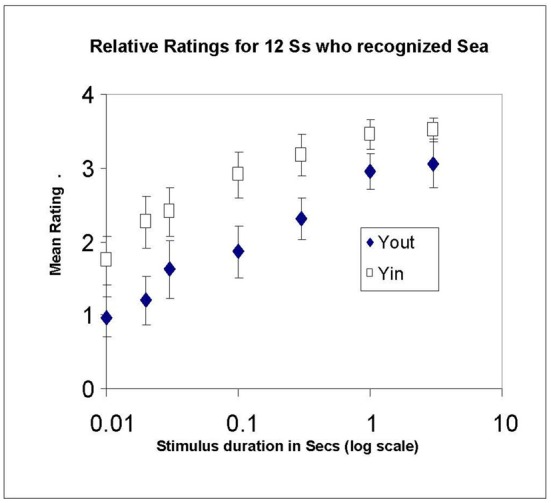
**Mean ratings in Experiment 3, made relative to a maximum of 4 for steady-state viewing of each illusory stimulus (Yin and Yout), plotted against stimulus duration in log s**. Only recognizers were run. Error bars show ±1 standard error of each mean.

Mean ratings are plotted in Figure 5, again on a log time basis to avoid compressing the data at short stimulus durations. Linear regressions showed the typical fast growth over stimulus durations from 0.01 to 0.1 s, namely, 0.85/100 ms for Yout, and 1.05/100 ms for Yin (*r*^2^ = 0.73 and 0.81, respectively). Growth was much slower from 0.3 to 5 s, at 0.11/s for Yin and 0.23/s for Yout. Growth patterns for Yin and Yout were generally similar. It is also clear that the illusion in Yin was stronger than in Yout, the average rating for Yin (2.78) exceeding that for Yout (2.00). The asymptotic rating, expected to be 4 at the longest duration for both conditions, only reached 3.5 for Yin and 3.0 for Yout at the longest duration used (5 s). In hindsight, we should have included an even longer duration to make quite certain that 4 was eventually reached, as per instructions.

To characterize the growth of the watercolor effect during the early period when the illusion is growing rapidly, we asked at what time does the effect reach half-way between the rating at 0.01 s and that at 0.1 s? Linear time axes were used to interpolate the half-way point. This calculation removes any effect of an overall difference in strength. Table 2 shows the mean ratings at 0.1 and 0.01 s, the difference between them (the “Effect” column), the half-way rating magnitude (Rating at 0.1 s minus half the Effect), and the half-way times (the time to reach the half-way magnitude). The rows show these values for each configuration (Yin, Yout) and recognition (did, didn't), in Experiments 2 (Yin-2-did, Yin-2-didn't, etc.) and 3 (Yin-3-did, etc.).

**Table 2:** **Estimates of the time in secs to reach 50% of the illusion as rated at 0.1 s**.

**Case**	**Rate:0.1 s**	**Rate:0.01 s**	**Effect**	**Half-way**	**Secs**
Yin-2-did	3.302	1.957	1.345	2.629	0.052
Yin-2-didn't	2.993	1.510	1.483	2.252	0.075
Yout-2-did	2.217	1.524	0.693	1.871	0.025
Yout-2-didn't	1.679	1.042	0.637	1.361	0.028
Yin-3-did	2.906	1.739	1.167	2.323	0.025
Yout-3-did	1.865	0.969	0.896	1.417	0.026

In Experiment 2, those who recognized the Sea attained a faster growth in the illusion than those who did not: the mean half-way times were 0.052 s and 0.075 s respectively. Recognizers experienced both more illusion and a faster growth in the illusion than non-recognizers.

Averaged over recognition status, growth in Yin was slower than in Yout in Experiment 2 (the first 4 lines of Table 2): the half-way times are 0.063 s in Yin and 0.027 s in Yout. We asked whether this slower growth for Yin was also the case just for recognizers, as these were run in both Experiments 2 and 3. Their half-way times were indeed longer (0.052 s) in Yin than in Yout (0.025 s) in Experiment 2, but were the same in Yin (0.025 s) as in Yout (0.026 s) in Experiment 3 (the last two lines of Table 2). Thus these results are inconclusive and we can only conclude that the growth in illusion strength over the first 100 ms in Yin is either equal to, or slower than, that in Yout. A matching experiment, not dependent on the choice of rating scale anchor, may be needed to resolve this ambiguity.

## Experiment 4 (masking)

Geometrical illusions such as the Ponzo and Zoellner illusions develop microgenetically, as demonstrated by Reynolds (1978), who followed a brief (50 ms) presentation of the illusion-generating pattern by a 200 ms patterned backward mask. At short inter-stimulus intervals (ISIs), subjects reported the stimulus as it truly was; only at an ISI of 100 ms did the illusions reach their full extent. Reynolds concluded that perception of the parts occurred before their interaction could create the illusion evident in the whole. Kurylo (1997) presented a columnar array of dots to study Gestalt grouping, which was followed by a patterned mask. He argued that the dots must first be located before they can be grouped, so that at longer array-to-mask intervals, only grouping would be interfered with (at shorter ones, locations might also be lost). On this basis he identified the “completion time” as that interval at which interference was first observed. Mean times to complete grouping were 88 ms by proximity and 119 ms by alignment. These times suggest a minimum for the watercolor effect since regions cannot be filled in until their boundaries are grouped into a unit.

We wondered whether backward masking could also be used to study the microgenesis of the illusory wash. Might the observers see a gray field for a period before the wash “filled in,” or was the fill-in virtually instantaneous? We ran both recognizers and non-recognizers to discover whether any such effect might be modified by prior knowledge.

### Procedure

The stimulus duration was held constant at 50 ms, long enough for the illusion to have developed about half-way according to the earlier ratings. The 50 ms stimulus was followed, after a blank ISI consisting of the same gray 116 cd/m^2^ field, by a colored, textured mask (Figure 1, right-hand panel). The gray field retained the same luminance during the ISI as before the stimulus and after the mask to avoid brightness transients (as might be created by a blank—i.e., dark—ISI). The mask was completely effective when presented simultaneously with the stimulus, rendering the stimulus invisible. Unlike Reynold's line masks, which were chosen to disrupt edge interactions, our mask consisted of a texture of multiple colored dots from green through red, averaging to yellow. This mask was chosen to disrupt filling-in by color, rather than the processing of the bichromatic outline. The ISIs were 10, 20, 30, 50, 100, 150, 200, 300, and 500 ms, and were randomized over trials.

Participants again rated the intensity of the illusion on a four-point scale after each appearance of the stimulus. As in Experiment 3, ratings were relative; that is, the maximum rating of “4” was assigned to Yin and separately to Yout, when these stimuli were presented continuously. Half the participants were shown the Yin stimulus before the Yout one, and the remainder, the reverse. Each participant received 6 stimuli at each ISI for a total of 54 trials each.

### Results and discussion (masking)

**Figure 6:**
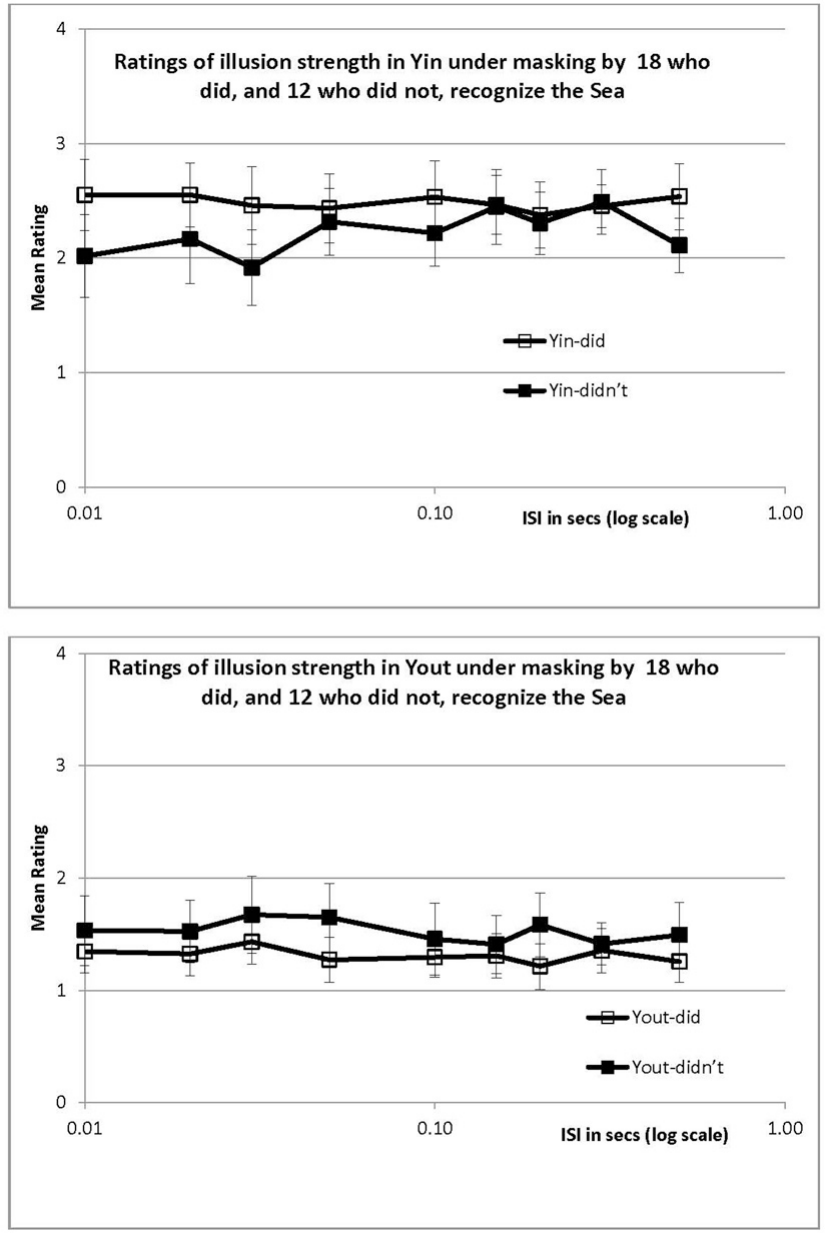
**Mean ratings in Experiment 4 (masking) of Yin (upper panel) and Yout (lower panel), for recognizers and non-recognizers, plotted against ISI in log s**. Ratings were relative, as in Experiment 3. Error bars show ±1 standard error of each mean.

Of the 30 participants, 18 did, and 12 did not, recognize the Sea. For both groups, the illusion reached its asymptote when the mask was presented immediately following the stimulus (an ISI of just 10 ms), and did not increase during the next 500 ms. This is shown by the flat curves in Figure 6 for the Yin configuration at the top and for Yout at the bottom; different symbols indicate recognition status. Regression analysis is hardly necessary but confirmed that ratings were essentially flat across ISI, with a mean change of −0.03/s (*r*^2^ values were less than 0.19 for each of the 4 plots in Figure 6, ns). The similarity of the data of those who did, and did not, recognize the Sea is marked; clearly the time course of masking, as revealed by these relative ratings, was independent of recognition status.

The steadily increasing growth of the illusion when stimulus duration is increased, seen in Experiments 2 and 3, is in clear contrast to the flat curves obtained when the ISI was increased in Experiment 4. Providing more processing time for the illusion by increasing the ISI had a quite different outcome compared to increasing processing time by increasing duration. In principle, increasing the ISI provides an opportunity for developing the illusion, as in Reynold's (1978) experiment, but this did not happen here. Rather, watercolor filling-in appears to happen simultaneously with the processing of the bichromatic contour, or perhaps just after but still during the 50 ms stimulus presentation. Additional post-exposure time has no effect.

## General discussion

The main outcome of the current research is that the watercolor illusion grows rapidly during the first 100 ms, but only when the stimulus is physically present; there was no further growth during the blank interval before the onset of a color-texture mask. This provides evidence that the watercolor effect forms rapidly, but requires a bottom-up signal, consistent with our understanding of the BCS/FCS model (Pinna and Grossberg, 2005), as boundary contour system (BCS) formation, which is bottom-up, immediately precedes filling in by the form system (FCS). However, two additional facts modify this picture. First, the illusion does grow, albeit much more slowly, if the exposure duration in increased from 0.1 to 10 s. (It is possible that this additional slow growth is related to monkey V1 “edge cells,” which progressively fill in color from about 0.3 to 8 s: von der Heydt et al., 2003). Secondly, those participants who did recognize the outline of the Sea experienced a faster growth to a higher level than those who did not recognize the Sea. One might have anticipated that the interaction with visual long-term memory that promoted the illusion in the recognizers would occur only after some delay, even in the BCS/FCS model. Yet the results show a clear advantage for recognizers even with 10 ms exposures, although the recognition effect does increase slightly with stimulus duration. Therefore the long-term memory interaction appears to be remarkably rapid. It is not known whether this interaction occurs afresh on each trial or reflects priming from previous trials, since only one watercolor stimulus (the Sea) was used throughout each experiment. Possibly primed signals are readied to guide microgenesis when stimuli are primed by an episodic form of long-term visual memory. A role for priming could be established by comparing repeated presentations of the same stimulus to presentations of novel stimuli on each trial.

A further outcome is that the watercolor effect can be measured quite precisely using a matching paradigm. This was not unexpected, as the illusory color is quite striking and does not fade with time. However, it is somewhat disturbing that previous measurements of the watercolor effect using matching to nonsense forms yielded a much smaller illusion, of 5.6% in (*u*′ *v*′) space (Devinck et al., 2005). One can postulate that the outline of the Sea was a nonsense stimulus for our non-recognizers. If so, recognition would have to more than triple the illusion from 5.6% (for non-recognizers) to 20% (for recognizers) to obtain our mean color shift of 13% for Yin in Experiment 1, given that half our participants recognized the Sea. Unfortunately we did not check for recognition in Experiment 1 and cannot ascertain if this is true, but the ratings do not suggest that recognition even doubles the effect, let alone triples it. Physical differences, such as in spatial area and contour complexity, may account for the remaining differences.

The possibility of measuring the watercolor illusion accurately means that it can be compared with other more traditional chromatic illusions. An obvious example is chromatic induction (Shevell and Wei, 1998). Since light scatter is one component of chromatic induction (Walraven, 1973) but not of the watercolor effect, a direct comparison of magnitudes is risky. However, Shevell and Wei (1998) were able to identify a source of chromatic induction from a neural signal for contrast at the edge of the test field using a method that controls for stray light. Ware and Cowan (1982) measured the extent of chromatic induction when test and inducer stimuli were alternating thin strips, each 6 min arc wide, similar to the lines in our watercolor stimulus. With light scatter and chromatic aberrations controlled, they found that a yellow inducer shifted a white test field chromaticity toward blue by 22% and 15% for each of two observers, in (*u*′, *v*′) space. This is somewhat comparable to our 13% effect in Yin, but much larger than the 5.6% effect found with irregular outline squares by Devinck et al. (2005). Future measurements will be needed to compare chromatic induction directly to the watercolor effect, using the same participants, adaptation conditions, and test stimuli, to discover whether these different illusory effects share any mechanisms or properties.

### Conflict of interest statement

The authors declare that the research was conducted in the absence of any commercial or financial relationships that could be construed as a potential conflict of interest.

## Footnotes

^1^For reference, *x* = *X/T* and *y* = *Y/T*, where *T* = *X* + *Y* + *Z* is the total light through the *X*, *Y*, and *Z* colorimetric filters. In the (*x*, *y*) system, the spectrum locus spans 0.04 < *x* < 0.72 and 0.01 < *y* < 0.82. CRT color displays cover about half of the total possible area, but this includes the watercolor illusion as this is desaturated and spans only the central region of the space. Transforming the (*x, y*) space into some other system is required to convey equal threshold intervals, relative receptor activations, or equal spacing of color appearances. At the prompting of a reviewer, we chose a uniform color space (*u*′, *v*′) so that distances in the space would be comparable in terms of threshold units, where *u*′ = 4*x*/*Q, v*′ = 9*y*/*Q*, and *Q* = 12 *y*− 2 *x* + 3. Data in (*u*′, *v*′) can be transformed back to (*X, Y, Z*) and thence to any other system, given that the luminance *Y* = 116 cd/m^2^.
